# Diagnosis of frontotemporal dementia: recommendations of the Scientific Department of Cognitive Neurology and Aging of the Brazilian Academy of Neurology

**DOI:** 10.1590/1980-5764-DN-2022-S103PT

**Published:** 2022-11-28

**Authors:** Leonardo Cruz de Souza, Mirna Lie Hosogi, Thais Helena Machado, Maria Teresa Carthery-Goulart, Mônica Sanches Yassuda, Jerusa Smid, Breno José Alencar Pires Barbosa, Lucas Porcello Schilling, Marcio Luiz Figueredo Balthazar, Norberto Anízio Ferreira Frota, Francisco Assis Carvalho Vale, Paulo Caramelli, Paulo Henrique Ferreira Bertolucci, Márcia Lorena Fagundes Chaves, Sonia Maria Dozzi Brucki, Ricardo Nitrini, Valéria Santoro Bahia, Leonel Tadao Takada

**Affiliations:** 1 Universidade Federal de Minas Gerais, Faculdade de Medicina, Departamento de Clínica Médica, Belo Horizonte MG, Brasil.; 2 Universidade Federal de Minas Gerais, Faculdade de Medicina, Grupo de Pesquisa em Neurologia Cognitiva e do Comportamento, Belo Horizonte MG, Brasil.; 3 Universidade de São Paulo, Faculdade de Medicina, Departamento de Neurologia, Grupo de Neurologia Cognitiva e do Comportamento, São Paulo SP, Brasil.; 4 Universidade Federal de Minas Gerais, Faculdade de Medicina, Departamento de Fonoaudiologia, Belo Horizonte MG, Brasil.; 5 Universidade Federal do ABC, Grupo de Estudos em Neurociência da Linguagem e Cognição, Programa de Pós-Graduação em Neurociência e Cognição, Centro de Matemática, Computação e Cognição, Santo André SP, Brasil.; 6 Universidade de São Paulo, Escola de Artes, Ciências e Humanidades, Gerontologia, São Paulo SP, Brasil.; 7 Universidade Federal de Pernambuco, Centro de Ciências Médicas, Área Acadêmica de Neuropsiquiatria, Recife PE, Brasil.; 8 Instituto de Medicina Integral Prof. Fernando Figueira, Recife PE, Brasil.; 9 Pontifícia Universidade do Rio Grande do Sul, Escola de Medicina, Serviço de Neurologia, Porto Alegre RS, Brasil.; 10 Pontifícia Universidade do Rio Grande do Sul, Instituto do Cérebro do Rio Grande do Sul, Porto Alegre RS, Brasil.; 11 Pontifícia Universidade do Rio Grande do Sul, Programa de Pós-Graduação em Gerontologia Biomédica, Porto Alegre RS, Brasil.; 12 Universidade Estadual de Campinas, Faculdade de Ciências Médicas, Departamento de Neurologia, Campinas SP, Brasil.; 13 Hospital Geral de Fortaleza, Serviço de Neurologia, Fortaleza CE, Brasil.; 14 Universidade de Fortaleza, Fortaleza CE, Brasil.; 15 Universidade Federal de São Carlos, Centro de Ciências Biológicas e da Saúde, Departamento de Medicina, São Carlos SP, Brasil.; 16 Universidade Federal de São Paulo, Escola Paulista de Medicina, Departamento de Neurologia e Neurocirurgia, São Paulo SP, Brasil.; 17 Hospital de Clínicas de Porto Alegre, Serviço de Neurologia, Porto Alegre RS, Brasil.; 18 Universidade Federal do Rio Grande do Sul, Faculdade de Medicina, Departamento de Medicina Interna, Porto Alegre RS, Brasil.; 19 Universidade Cidade de São Paulo, São Paulo SP, Brasil.

**Keywords:** Frontotemporal Dementia, Aphasia, Primary Progressive, Demência Frontotemporal, Afasia Progressiva Primária

## Abstract

“Frontotemporal dementia” (FTD) is a clinical syndrome characterized by the focal involvement of the frontal and/or temporal lobes. FTD has three clinical phenotypes: the behavioral variant and two linguistic subtypes, namely, non-fluent/agrammatic primary progressive aphasia (PPA-NF/A) and semantic PPA (PPA-S). FTD is the second most common cause of dementia in individuals under the age of 65 years. This article presents recommendations for the diagnosis of FTD in the Brazilian scenario, considering the three levels of complexity of the health system: primary health care, secondary and tertiary levels. Diagnostic guidelines are proposed, including cognitive testing, behavioral and language assessments, laboratory tests, and neuroimaging.

## RESUMO

A “demência frontotemporal” (DFT) é uma síndrome clínica, cujo denominador comum é o acometimento focal dos lobos frontais e/ou temporais. A DFT tem três fenótipos clínicos distintos: a variante comportamental e dois subtipos linguísticos, a saber, a afasia progressiva primária não-fluente/agramática (APP-NF/A) e a afasia progressiva primária semântica (APP-S). A DFT é a segunda causa mais comum de demência em indivíduos com idade inferior a 65 anos, após a doença de Alzheimer. O presente artigo apresenta recomendações para diagnóstico da DFT no cenário brasileiro, considerando os três níveis de complexidade do sistema de saúde: atenção primária à saúde e níveis secundários. São propostos protocolos de investigação diagnóstica abrangendo testagem cognitiva, avaliação comportamental, avaliação fonoaudiológica, exames laboratoriais e de neuroimagem.

## INTRODUCTION

Frontotemporal dementia” (FTD) is defined as a clinical syndrome whose common denominator is the focal involvement of the frontal and/or temporal lobes. FTD has three distinct clinical phenotypes: the behavioral variant, and two linguistic subtypes, namely, non-fluent/agrammatic primary progressive aphasia (PPA-NF/A) and semantic PPA (PPA-S). Recently, the existence of a fourth subtype, the so-called right temporal variant, has been considered. The behavioral variant (bvFTD) is the most common phenotypic presentation of FTD.

In turn, “frontotemporal lobar degeneration (FTLD)” refers to the histopathological substrate of FTD. The types FTLD-Tau and FTLD-“transactive response DNA-binding protein with Mr 43 kDa” (TDP-43) are the most common ones^1^. Other subtypes, such as FET family proteins, FUS (fused in sarcoma) and EWS (Ewing’s sarcoma protein), are less frequent.

Thus, “FTD” should be used for clinical descriptions and phenotypes, whereas “FTLD” should be used to describe the histopathological classification and not to refer to the clinical syndrome^2^.

International studies indicate that the prevalence and incidence of FTD compose 15-22 cases/100,000 and 1.2-4.1 cases/100,000 population, respectively, being higher in the 45-64 age group^3^ ^),(^ ^4^. There are no epidemiological studies that have specifically investigated the prevalence of FTD in Brazil, but epidemiological surveys on dementias indicate a prevalence of 0.18% among individuals over 65 years old^3^.

Such indices make FTD the second most common cause of presenile dementia, after Alzheimer’s disease (AD). Indeed, the majority of FTD cases are of young onset, but it is now recognized that up to 30% of cases start after 65 years old^2^.

This article presents recommendations for the diagnosis of FTD in the Brazilian scenario, considering the three complexity levels of the health system: primary health care, secondary and tertiary levels. Diagnostic guidelines are proposed, covering cognitive testing, behavioral and language assessments, laboratory tests, and neuroimaging. The right temporal variant, whose clinical definition is still in debate^5^ and is not part of the latest diagnostic consensus^6^ ^),(^ ^7^, is not addressed in this article.

## DIAGNOSIS

The diagnostic criteria for FTD, both for the behavioral^6^ ^)^ and the language variants^7^, were established by international expert consensus.

The diagnosis of bvFTD is based on the identification of progressive cognitive-behavioral decline^6^. The current criteria determine three levels of diagnostic reliability (Table 1): (I) possible diagnosis, for patients presenting characteristic cognitive-behavioral alterations, but have neither typical neuroimaging alterations nor manifest functional decline; (II) probable diagnosis, for patients who, in addition to characteristic cognitive-behavioral manifestations, have impaired autonomy and evidence of frontotemporal involvement on structural or functional neuroimaging; and (III) definitive diagnosis, when histopathological changes are observed on brain biopsy or on post-mortem examination, or patients with evidence of pathogenic mutation.

**Table 1:** Diagnostic Criteria for Frontotemporal Dementia (behavioral variant) - adapted from Rascovsky et al. (2011).

Frontotemporal dementia: behavioral variant
Progressive deterioration in behavior and/or cognition, evidenced by observation or clinical history (requires an informant).
**Possible diagnosis**
Must meet at least 3 of the 6 criteria listed below:
(I) behavioral disinhibition (socially inappropriate behavior; impulsiveness; loss of social rules or decorum);
(II) apathy or inertia;
(III) loss of empathy/sympathy (decreased affective resonance to the needs and feelings of others; diminished social interest, reduced interpersonal “heat”);
(IV) perseverative, stereotyped, or compulsive/ritualistic behaviors;
(V) hyperorality and dietary changes (alteration of food preference, increased consumption of alcohol or cigarettes, oral exploration of objects);
(VI) neuropsychological profile with executive deficits and relative preservation of episodic memory and visuospatial functions.
**Probable diagnosis**
Must meet criteria for possible FTD and have:
(I) Significant functional decline, demonstrated in specific inventories; and
(II) Evidence of typical FTD alterations on neuroimaging examination (atrophy or hypometabolism or hypoperfusion in frontotemporal regions).
**Definite diagnosis**
Must meet criteria for possible or probable FTD and have:
(I) Pathological confirmation of frontotemporal lobe degeneration; or
(II) Evidence of causative genetic mutation.

According to the same consensus^6^, for possible or probable diagnosis, the patient must meet at least three of the following criteria: (I) early disinhibition; (II) apathy or early inertia; (III) early loss of empathy/sympathy; (IV) perseverative, stereotyped, or early compulsive/ritualistic behavior; (V) hyperorality and dietary changes; and (VI) neuropsychological profile with executive dysfunction and relative preservation of episodic memory and visuospatial abilities. The symptom is early if it occurs within the first three years after the onset of symptoms.

PPA, in turn, is a clinical syndrome characterized by a language disorder of insidious onset and progressive course, which affects the functioning of the language network in the language-dominant temporal and frontal lobes^7^ ^),(^ ^8^. In most cases, there is predominant involvement of the left hemisphere, with rare exceptions (crossed aphasia and/or conditions that start with neurodegeneration on the right hemisphere and with initial symptoms of prosopagnosia and/or visual agnosia) ^(^ ^9^. Language disorders involve one or more levels of linguistic processing (phonological, semantic, syntactic) and are associated with cognitive (i.e., speech apraxia) and/or motor (dysarthria) speech alterations. In addition, language impairment impacts communication skills; thus, functional deficits vary depending on the language demands related to professional occupation and daily activities, and to the cognitive resources and strategies that patients use to compensate for the deficits, as well. Environmental factors can also mitigate or enhance the functional impairment^10^.

Gorno-Tempini et al. (2011) suggested unifying the nomenclature and defined criteria for the syndromic diagnosis of PPA and its three main variants, in terms of clinical manifestations, neuroanatomical and neuropathological correlates. PPA-NF/A (non-fluent/agrammatic) and PPA-S (semantic) are part of the clinical syndrome of FTD. On the contrary, the logopenic subtype of PPA (PPA-L) is considered an atypical presentation of AD. It should be noted, however, that about 1/3 of the patients have mixed conditions or do not fit the criteria for these variants^11^ ^),(^ ^12^. Thus, although the diagnosis of PPA can be accurately distinguished from bvFTD and/or other dementia conditions, classification into variants requires speech and language examination by experts. In the present consensus, we have chosen the terms PPA-S, PPA-NF/A and PPA-L. Other terminologies are also used, including: “semantic variant of PPA,” “non-fluent variant of PPA,” and “logopenic variant of PPA.”

According to Gorno-Tempini et al. (2011) ^(^ ^7^, the clinical diagnosis of PPA requires fulfilling three criteria: (1) the most prominent clinical feature is language difficulty; (2) language difficulties are the main cause of functional impairment (difficulty in communication); and (3) aphasia must be the most prominent deficit at the beginning of the condition. Furthermore, the pattern of deficits must not be explained by another neurological or psychiatric disorder, and patients must not initially present significant behavioral disturbances or other cognitive impairments. However, deficits in other cognitive functions appear in the neuropsychological assessment, especially in cognitive skills that share neuroanatomical correlates with the language network (such as verbal immediate memory; numerical and calculation skills; ideomotor praxis). However, these deficits should be milder compared to the language deficit. In addition to the clinical level, the consensus predicts two other diagnostic levels: one supported by neuroimaging exams and another supported by histopathological findings.

**Figure 1:**
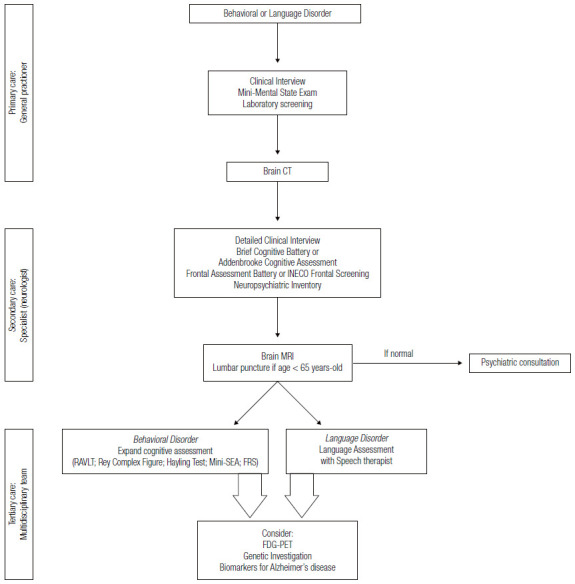
Diagnostic procedures for frontotemporal dementia

**Figure 2:**
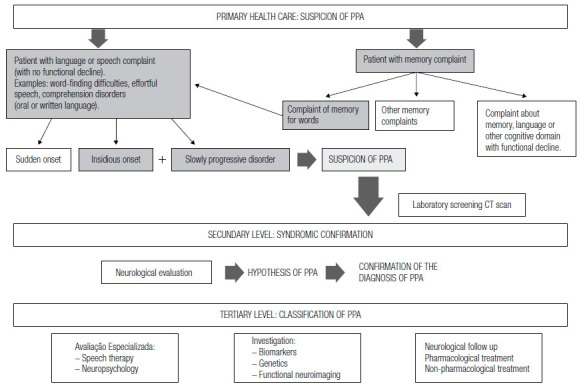
Procedures for the diagnosis of Primary Progressive Aphasia

Next, we propose ways to carry out the diagnostic investigation of FTD (all subtypes) at different levels of health care (Figures 1 and 2).

### Primary health care

#### Clinical assessment (bvFTD and PPA)

In primary health care, the patient will be initially evaluated by a general practitioner, who must carry out a careful anamnesis in order to identify a condition of cognitive-behavioral decline or progressive language disorder and then refer the patient to the appropriate workup. Therefore, it is important to describe how symptoms appear. Due to its degenerative nature, the onset of FTD symptoms is usually insidious and progressive, unlike vascular conditions, in which the “stepwise deterioration” of symptoms is more common.

Considering the clinical suspicion of cognitive-behavioral decline, the physician must actively inquire about psychiatric (past admission to psychiatric institutions) and infectious (syphilis, HIV) antecedents and about the use of psychotropic drugs that may interfere with cognitive-behavioral functioning. Mood symptoms and maniform manifestations should also be always investigated, as they may indicate a psychiatric cause for the patient’s clinical condition. On physical examination, the physician should perform a brief neurological examination, looking for focal deficits that suggest stroke or expansive intracranial lesions.

Regarding progressive language disorders, patients and family members seek medical care due to complaints of memory or specific language and/or speech problems (Figure 2). When faced with the memory complaint, it is important to differentiate which memory problem the patient and family refer to. Often, patients having difficulty finding words and forgetting the names of people and objects (essentially linguistic alterations) report that they have memory failures, but these are not episodic memory alterations (common in AD), but rather word memory (lexical access difficulties and anomie), suggesting language impairment. In some cases, it is possible that the memory complaint is related to the gradual loss of knowledge of the meaning of words and concepts (for example: having doubts about what the words mean, which is a symptom of impaired verbal semantic memory).

Initial language complaints may be diverse. The most frequent are lexical access difficulties and anomia. However, there are also reports of exchange of words, exchange of phonemes, stuttering, slowing down of speech production, language comprehension difficulties, failures in reading or writing and so forth. It is important to investigate whether the patient and/or informant (e.g., family member) notices an insidious worsening of language in relation to premorbid performance. There is great heterogeneity of language in the population, depending on education, reading and writing habits, and occupation. It may be tough to detect difficulties in individuals who, throughout life, had poor language skills (for example, writing errors, low reading fluency, impoverished vocabulary). Therefore, in some cases, it may be necessary to conduct a longitudinal follow-up to identify the progression of the condition. Clinical history is fundamental for the diagnosis of PPA. Speech-language disorders, in the initial phase, do not significantly impact functional activities, and some patients even keep their work activities.

#### Cognitive and behavioral assessments

The assessment of cognitive performance represents an important component of the diagnostic procedures of FTD. Cognitive changes in bvFTD can be heterogeneous, but the diagnostic criteria of Rascovsky et al. (2011) suggest that the neuropsychological profile should include executive dysfunction, relative preservation of episodic memory and of visuospatial functions. However, in the early stages of the disease, especially among people with a high level of education, executive dysfunction may not be evident^13^. Additionally, other diseases (including AD) can lead to executive dysfunction^14^. Another challenging factor is that patients with bvFTD can also present episodic memory deficit, in a pattern similar to that observed in AD^15^ ^),(^ ^16^. Even so, documenting the cognitive profile suggested in the international criteria^6^ is important for the diagnosis of bvFTD. According to this perspective, identifying a reduction in global cognitive efficiency and executive dysfunction of magnitude greater than episodic memory deficits may suggest bvFTD.

In primary care, complaints from patients and their families suggesting the presence of cognitive deficits should be carefully analyzed, especially those indicating difficulties in planning and organizing activities. The Mini-Mental State Examination (MMSE) ^(^ ^17^ ^),(^ ^18^ should be applied for the global assessment of cognition, and the score should be interpreted in relation to the individual’s educational level. Patients with bvFTD are likely to lose points in mental calculation and the command sub-items (due to attention deficit); orientation in time and space are usually preserved^19^, as the drawing. Impairment in the MMSE may suggest the presence of dementia, which should be confirmed in further evaluations in secondary care.

Social cognition involves processing information that is relevant to social interaction. In bvFTD, social cognition may be altered, as the patient may fail to understand what others are thinking or feeling. The clinician should ask the caregiver whether the patient understands the problems and concerns of those around them (cognitive empathy) and whether they care, suffer, or rejoice in what happens to others (emotional empathy). Questions can be asked about their ability to socialize and changes in their moral rules. From this perspective, primary care physicians should inquire caregivers and observe patients regarding typical behavioral changes of FTD, in addition to other neuropsychiatric manifestations, such as delusions and hallucinations, which may suggest primary psychiatric disorders.

The performance of patients with PPA on the MMSE will depend on the intensity of the aphasia, as it is a test that is highly dependent on language. MMSE results may overestimate cognitive decline, as cognitive functions such as temporal orientation and memory are assessed by language. On the other hand, the language deficit can be underestimated, since the MMSE linguistic tasks have low complexity to assess the production and comprehension of words and sentences. Thus, initial cases of PPA can obtain scores within the expected range for their schooling.

#### Laboratory investigation

Metabolic and/or infectious disorders (renal or liver failure, hypothyroidism, neurosyphilis and HIV infection, and so on) that cause neuropsychiatric manifestations can be ruled out through blood tests. Thus, all suspected cases of FTD (regardless of the phenotypic presentation) should undergo laboratory investigation to screen for reversible causes of cognitive-behavioral decline^20^: blood count, vitamin B12, folic acid, liver, kidney and thyroid functions, protein electrophoresis, and anti-HIV serology.

#### Neuroimaging investigation

In primary health care, CT scan can be a useful procedure as an initial propaedeutic. This exam evaluates the presence of frontotemporal lesions (e.g., tumors, hematomas), ventricular dilatation or cerebrovascular lesions that are associated with behavioral symptoms. Computed tomography in bvFTD usually shows asymmetric increase in sulci and fissures in the frontal and/or temporal lobes. These findings, however, can only be observed when the disease is at a more advanced stage. In the linguistic variants, frontoinsular atrophy in the dominant hemisphere can be observed, in the case of the PPA-NF/A variant, and atrophy of temporal poles, in the case of the PPA-S.

### Secondary level

#### Clinical assessment

At the secondary level, the patient will be evaluated by a neurologist, who must carry out a thorough neurological examination, preceded by a detailed anamnesis, which aims to recapitulate the main elements of clinical and family history. The neurologist should actively look for signs of parkinsonian syndrome, in addition to changes in oculomotricity (conjoined down-gaze palsy), which may evoke progressive supranuclear palsy. Similarly, signs of asymmetric muscle atrophy, fasciculations and pyramidal signs (Babinski and Hofmann signs) should be looked for to identify signs of motor neuron disease. The neurologist should also look for the presence of primitive signs (grasping, glabellar, snouting) that suggest severe frontal involvement.

#### Cognitive and behavioral assessments

At this level of care, the assessment of cognition must be comprehensive and evaluate the main cognitive domains. The application of the Brief Cognitive Screening Battery (BBRC) is recommended^21^ ^),(^ ^22^. The BBRC includes the Figure Memory Test, which requires naming and recalling ten figures to assess episodic memory. In this battery, executive functions are investigated with the Animal Verbal Fluency Test and the Clock Drawing Test, which are followed by the delayed recall of the ten figures previously presented (after 5 minutes). Special attention should be paid to the delayed recall (cut-off score ≤ 5 pictures), as it is a marker of episodic memory impairment. The BBRC can be used in populations with different educational backgrounds^23^. Slowness in naming, lack of responses and/or phonological/semantic errors may indicate difficulties in understanding or expressing the language. Episodic memory is preserved in the early stages of PPA. Patients usually perform well in the recognition task, as it does not require oral recall.

The Addenbrooke Cognitive Examination - Revised (ACE-R) ^(^ ^24^ ^),(^ ^25^ is also an excellent tool for the global assessment of cognition. The ACE-R includes the MMSE. Additionally, it provides individualized scores for attention and orientation, episodic memory, verbal fluency, language, and visual-spatial skills. The scores for the verbal fluency and language domains can be especially relevant for the diagnosis of bvFTD and PPA, respectively.

The Frontal Assessment Battery (FAB) ^(^ ^26^ ^),(^ ^27^ ^)^ and the INECO Frontal Screening (IFS) ^(^ ^28^ can identify executive dysfunction and help detect patients with bvFTD. However, these instruments may fail in the differential diagnosis of dementia subtypes^29^.

Regarding social cognition, the physician should ask the caregiver if the patient understands the problems and concerns of the people around them (cognitive empathy) and if they care, suffer, or are happy with what happens to others (emotional empathy). Questions about their social skills and changes in their moral rules can also be asked.

The short version of the Neuropsychiatric Inventory (NPI) ^(^ ^30^ ^),(^ ^31^, the NPI-Q^32^, can be used in the investigation of neuropsychiatric symptoms. The NPI-Q can detect the behavioral symptoms of bvFTD. The NPI-Q has been validated for the Brazilian population^33^. The Frontal Behavioral Inventory can also be used^34^.

#### Laboratory investigation

At the secondary level, the physician must review all laboratory tests to screen for reversible causes of dementia. For pre-senile patients, it may be necessary to include tests of autoimmune diseases, such as vasculitis and systemic lupus erythematosus, depending on the patient’s clinical context.

For patients suffering from the condition before turning 65 years old, or for those with a rapidly progressive decline, lumbar puncture is mandatory, to rule out inflammatory and/or infectious causes of dementia.

#### Neuroimaging investigation

**Figure 3:**
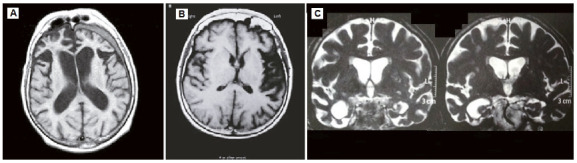
A. Focal atrophy of frontotemporal regions in the behavioral variant of Frontotemporal Dementia; B. Atrophy of the inferior frontal gyrus of the dominant hemisphere in primary non-fluent/agrammatic progressive aphasia (PPA-NF/A); C. Focal temporal pole atrophy in semantic primary progressive aphasia (PPA-S).

In secondary care, magnetic resonance imaging (MRI) of the brain should be performed. On MR images, an increase in sulci and fissures can be observed, with frontotemporal predominance (Figure 3). bvFTD is characterized by early atrophy of frontotemporal regions, affecting the anterior cingulate and orbitofrontal cortex^35^ ^),(^ ^36^. Hippocampus may be atrophied in bvFTD to a similar degree to that seen in AD^37^. Some atrophic patterns may suggest FTD associated with genetic mutations: pronounced bitemporal atrophy occurs in patients with a *MAPT* mutation; markedly asymmetric frontotemporal atrophy is common in patients with a progranulin (*GRN*) mutation^38^. Extensive white matter lesions can also occur in patients with progranulin mutation^39^. As disease progresses, atrophy gets more pronounced in the frontotemporal lobes, with relative preservation of the posterior regions. In PPA-NF/A, atrophy of the inferior frontal gyrusof the dominant hemisphere is detected, whereas in PPA-S there is typically focal atrophy of the temporal pole, uni- or bilaterally.

MRI also allows the identification of hypersignal on T2-weighted and FLAIR sequences, which could suggest vascular dementia or leukoencephalopathy.

### Tertiary level

#### Clinical Evaluation

At the tertiary level of health care, the patient will be assisted by a multidisciplinary team. The recommended clinical assessment consists of anamnesis, clarifying previously obscure points and confirming previous data. The clinical-neurological examination must follow the guidelines described for the secondary health level.

#### Cognitive and behavioral assessments

Behavioral variant (bvFTD)

At this level of care, it may be useful for diagnostic purposes to characterize the patient’s neuropsychological profile, with specific and detailed parameters for each domain of cognition. Qualitative observation of the patient’s behavior during the assessment may be especially relevant. The presence of unusual behaviors and strategies during testing, such as impulsiveness, behavioral rigidity, ritualized or obsessive behaviors, and repetitive speech, can support the diagnosis of bvFTD^40^.

Next, we suggest instruments composing a minimal neuropsychological battery. This proposal can be expanded, according to available time and expert facilities:

- Verbal episodic memory - Rey Auditory-Verbal Learning Test^41^. Scores within the expected range for age and education, or minor impairment, may be compatible with a diagnosis of bvFTD;
- Visual episodic memory - Rey Complex Figure^42^, which requires the individual to copy a complex geometric figure, which is later drawn using visual memory. The copy is used to assess visual-constructive skills and the subsequent recall of the figure is a measure of visual episodic memory;
- Attention and Executive Functions - Trail Making Test A and B assess visual attention and shifting, respectively; Wechsler Adult Intelligence Scale (WAIS-III) Forward and Backward Digit Span Test assess auditory attention and working memory, respectively; Wisconsin Card Sorting Test assesses working memory and mental flexibility. These cognitive parameters may be especially important to characterize executive dysfunction, which is part of the formal criteria of bvFTD^6^. However, they may not discriminate between types of dementia^43^;
- Inhibitory Control - The Hayling Test assesses the individual’s ability to complete sentences with words that make sense or with words that prevent the logical sense of the sentence. The test could help differentiate bvFTD from other dementias such as AD, but results are inconclusive^44^. Likewise, the Stroop test may not be discriminative^43^ ^)-^;
- Processing Speed - WAIS-III Digit Symbol Substitution test assesses visual attention and processing speed, which, if altered, can affect performance in other domains of cognition^45^;
- Visuospatial Functions - Rey Complex Figure. The copy of the figure can be used as a parameter to assess planning and visuoconstructive skills; WAIS-III Block Design also assesses visual-constructive skills and is a timed task; the Visual Object and Space Perception (VOSP) test can be used to assess visual-perceptual abilities^46^. According to current criteria, patients with bvFTD have unimpaired visuospatial capabilities^6^. For example, in the Rey Complex Figure test, patients with bvFTD tend to perform well, and errors are usually related to failures in organization and planning, while patients with AD commit visuospatial errors^43^;
- Language - the Boston Naming Test assesses the ability to perceive, interpret and name 60 common figures. The importance of using the version adapted for Brazil is highlighted^47^. The WAIS-III Vocabulary test may also be helpful^45^;
- Social cognition - Although not included in current criteria for diagnosing bvFTD, a growing body of evidence suggests that tests of social cognition may be useful for the differential diagnosis between bvFTD and other dementias, such as AD^48^ ^),(^ ^49^. Studies have shown that the Facial Emotion Recognition Test (FERT) and the *Faux-Pas* Test (which assesses theory of mind) are useful in the differential diagnosis between bvFTD and AD^48^ ^),(^ ^49^. The short version of the Social and Emotional Assessment (Mini-SEA) ^(^ ^50^- consisting of the FERT and the *Faux-Pas* test - is an efficient instrument to differentiate bvFTD from AD, regardless of executive dysfunction^51^, apathy^52^ and episodic amnesia^53^. It also differentiates bvFTD from depressive disorder^50^, and it is also recommended to distinguish bvFTD from other primary psychiatric disorders^54^. Despite the lack of formal validation of the Mini-SEA in Brazil, its clinical validity among Brazilian patients has already been demonstrated in research^51^ ^),(^ ^52^. There is a cross-cultural study of FERT with normative data for the Brazilian population^55^ and a cross-cultural adaptation of the *Faux-Pas* test, with the assessment of its psychometric properties^56^;
- The Frontotemporal Dementia Rating Scale (FRS) ^(^ ^57^ is a useful scale for staging the disease. It has been validated for use in Brazil^58^.

Concerning the investigation of behavioral changes, the Frontal Behavioral Inventory (FBI) ^(^ ^59^ ^),(^ ^60^ ^)^ is useful in the differential diagnosis between bvFTD and other dementias and has good psychometric properties^60^ ^)-(^ ^62^. Recently, the FBI has been recommended for the differential diagnosis between FTD and Primary Psychiatric Diseases^54^. There is no validation of this scale in Brazil, but there is a translation^34^.

The DAPHNE scale^63^ is based on the bvFTD diagnostic criteria 6 and on the FBI items. There is only one study to evaluate its application in the differential diagnosis between bvFTD and AD (frontal variant), with good clinical accuracy^64^. There is no Brazilian validation or translation of this scale, but it appears to be promising.

For an accurate differential diagnosis between FTD and PPD, a formal evaluation by a psychiatrist with experience in degenerative dementias is recommended.

Linguistic variants (PPA-NF/A and PPA-S)

The investigation and characterization of language and speech impairments should encompass both spontaneous conversation and the testing of specific language skills, including phonological, lexical/semantic and syntactic levels.

Spontaneous conversation, in addition to the elaboration of speech from a picture, provides information on oral fluency and speech motor capacity. Patients with PPA-NF/A often have reduced oral production; they may manifest articulatory and/or syntactic errors, slow speech rhythm, and difficulty in lexical access. Patients with PPA-S are fluent, with preservation of the syntactic structure, but with difficulties in lexical access; they also manifest semantic and verbal paraphasias, and frequently use generic words (“things,” for example), circumlocutions and compensatory gestures^7^ ^),(^ ^65^.

Variable degree of anomia is a symptom common to all PPA subtypes; for this reason, visual confrontation naming tests should always be used. Not only the final score, but the types of paraphasias, are important for the diagnosis of the PPA variant. In PPA-S, the difficulty in naming is accompanied by semantic impairment, with frequent semantic paraphasias, use of circumlocution and supracategorization (name a dog as an animal, for example), while in PPA-NF/A the deficit comes from failure to access the lexicon. In PPA-S, it is possible that the patient does not identify the visual stimulus. So, in addition to paraphasias, there are answers such as “what is this?”, “I don’t understand this one.”

In PPA-S, performance on specific tests of word comprehension is necessarily impaired, and performance on object knowledge tests may be altered, especially on less familiar items; patients with PPA-NF/A have satisfactory results in both tasks. Conversely, the comprehension of complex sentences is frequently altered in PPA-NF/A, but it is generally preserved in PPA-S.

Repetition of sentences with different lengths and complexities is used to investigate the phonological loop of working memory. Patients with PPA-NF/A may have repetition difficulties due to praxis problems. Individuals with PPA-S, however, show no difficulty in this activity.

The analysis of the type of errors in reading and writing tasks of regular, irregular words and pseudowords makes it possible to differentiate patients with semantic impairment from those with phonological difficulties. Patients with semantic deficits manifest dyslexia and/or surface dysgraphia, in which irregular words are read and/or written with oral support. The production of sentences and written texts also contributes to the recognition of syntactic difficulties. Patients with PPA-NF/A may present dyslexia and/or phonological dysgraphia.

Motor and praxis assessment of speech, including diadochokinesia tests, helps the identification of dysarthria and/or speech apraxia. Speech apraxia is a disturbance in the planning and programming of sensorimotor commands necessary to perform articulatory movements. It is different from dysarthria, which compromises the motor system. Speech apraxia is a primarily articulatory disorder, and prosody impairment may occur secondarily. As it is also responsible for changes in speech sounds, it can be misdiagnosed as phonological impairments in oral emission.

Performance on language tasks is significantly impacted by education, occupation, and language experiences (reading and writing habits, bilingualism, participation in social activities and hobbies involving language). Research in Brazil has advanced to validate and obtain standards for language tests. For a review of available instruments to assess adult language, see Parente et al. ^(^ ^66^.

Due to neuropathology, motor deficits are often associated with cognitive and behavioral conditions. Thus, the speech therapy assessment should investigate difficulties related to swallowing.

#### Laboratory investigation

For patients who undergo lumbar puncture, the investigation of CSF biomarkers (beta-amyloid peptide, Tau and P-Tau) is useful to rule out AD, in cases of difficult differential diagnosis^67^ ^),(^ ^68^. However, it should be noted that there are methodological issues that must be considered when indicating and interpreting the dosage of biomarkers: the absence of established universal reference values, analytical variability, the significant occurrence of false-positive results in patients over 70 years old, the difficulty of performing the exam, and the onerous cost^69^.

Unlike the existing biomarkers for AD, there are currently no specific biomarkers of pathophysiological changes associated with FTLD. The light chain neurofilament (NFL) has been intensively researched in degenerative dementias, including FTD. NFL reflects axonal damage and is increased in FTD, as in other neurodegenerative diseases, such as AD. NFL measurement is useful in differentiating FTD from other non-degenerative conditions such as primary psychiatric disorders^68^. NFL dosage also appears to correlate with disease severity^68^. Despite its potential interest, the measurement of NFL is not currently available for use in Brazil.

Most cases of FTD are sporadic^70^. However, in about 40% of cases, a family history of dementia is identified. In approximately 10 to 15% of patients, an autosomal dominant transmission pattern can be found, such as mutations in the *MAPT* gene (“microtubule-associated protein tau”), in the progranulin (*GRN*) gene or the expansion. In Brazil, we found mutations in the progranulin gene in about a third of familial cases, while *c9orf72* expansions and pathogenic variants in *MAPT* were found in about 10% of familial cases each^70^. Knowledge about monogenic forms of FTD has grown significantly over the last ten years and, currently, more than twenty genes with pathogenic variants are known as genetic causes of FTD (many of them also cause amyotrophic lateral sclerosis [ALS]).

The most frequent clinical presentations in pathogenic variants of the progranulin gene are bvFTD (about 40% of cases in a multicenter study), PPA-NF/A in 9%, and corticobasal syndrome in 4%. About 8% of patients with this mutation have been diagnosed with dementia of the Alzheimer type. The syndromes most frequently associated with *c9orf72* expansions are bvFTD (31%), FTD with ALS (11%) and ALS (20%). In cases of pathogenic variants in the *MAPT* gene, the most frequently described clinical syndromes are bvFTD (44%), progressive supranuclear palsy (4%), and Parkinson’s disease (5%)^71^.

Currently, the genetic investigation of familial FTD cases is done by requesting the search for pathogenic variants in gene panels associated with FTD or, preferably, given the wide range of possible genes related to the disease in a given family through exome sequencing. Importantly, the search for *c9orf72* expansion cannot be evaluated by exome analysis and a separate analysis is needed to identify the presence of this mutation. In cases of FTD with ALS, the search for *c9orf72* expansion may even precede exome sequencing. Of note, even in familial cases, the exome sequencing and expansion search in *c9orf72* may be negative, and a mutation may not be identified. In a recent multicenter study, pathogenic variants in the three major genes (*GRN, MAPT, c9orf72*) were only identified in 61% of familial cases^72^.

#### Neuroimaging investigation

At the tertiary level, MRI is also used as the main diagnostic imaging method. However, in the early stages of the bvFTD, structural changes may be subtle or even absent. In this context, the use of functional neuroimaging (cerebral perfusion scintigraphy and, mainly, positron emission tomography with fluorodeoxyglucose) may allow the identification of alterations that suggest a neurodegenerative process (hyperfusion or hypometabolism), before the atrophy is undoubted. Functional neuroimaging exams may show metabolic or perfusion dysfunction in the prefrontal cortex and temporal poles, depending on the clinical type of FTD. Positron emission tomography has better diagnostic accuracy than scintigraphy.

Currently, there are molecular neuroimaging methods of positron emission tomography, which allow the detection of cerebral beta-amyloid (such as PET with Pittsburgh Compound B, or PiB). Although it does not help in the diagnosis of bvFTD, it is a method that allows the diagnosis of the frontal (or dysexecutive) variant of AD^73^, which is an atypical presentation of the disease and manifests with apathy, emotional blunting, depressive symptoms, and episodic memory deficit. There are also radiotracers that bind to the tau protein; however, so far, these tracers seem to be better at detecting tau deposits related to AD.

In conclusion, the diagnosis of different variants of FTD is mainly based on clinical interviews and the assessment of cognitive, linguistic and behavioral aspects. Complementary tests provide valuable information to support the diagnosis and to rule out causes that may be similar to FTD conditions. The advent of new biological markers may provide greater diagnostic accuracy in the years to come.
